# Assisting nurses in care documentation: from automated sentence classification to coherent document structures with subject headings

**DOI:** 10.1186/s13326-020-00229-7

**Published:** 2020-09-01

**Authors:** Hans Moen, Kai Hakala, Laura-Maria Peltonen, Hanna-Maria Matinolli, Henry Suhonen, Kirsi Terho, Riitta Danielsson-Ojala, Maija Valta, Filip Ginter, Tapio Salakoski, Sanna Salanterä

**Affiliations:** 1 Department of Future Technologies, University of Turku, Vesilinnantie 5, Turku, 20500 Finland; 2 University of Turku Graduate School, University of Turku, Hämeenkatu 4, Turku, 20500 Finland; 3 Department of Nursing Science, University of Turku, Joukahaisenkatu 3-5, Turku, 20520 Finland; 4 Turku University Hospital, Kiinamyllynkatu 4-8, Turku, 20521 Finland

**Keywords:** Patient care documentation, Nursing documentation, Electronic health records, Text classification, Natural language processing, Neural networks, Model interpretation

## Abstract

**Background:**

Up to 35% of nurses’ working time is spent on care documentation. We describe the evaluation of a system aimed at assisting nurses in documenting patient care and potentially reducing the documentation workload. Our goal is to enable nurses to write or dictate nursing notes in a narrative manner without having to manually structure their text under subject headings. In the current care classification standard used in the targeted hospital, there are more than 500 subject headings to choose from, making it challenging and time consuming for nurses to use.

**Methods:**

The task of the presented system is to automatically group sentences into paragraphs and assign subject headings. For classification the system relies on a neural network-based text classification model. The nursing notes are initially classified on sentence level. Subsequently coherent paragraphs are constructed from related sentences.

**Results:**

Based on a manual evaluation conducted by a group of three domain experts, we find that in about 69% of the paragraphs formed by the system the topics of the sentences are coherent and the assigned paragraph headings correctly describe the topics. We also show that the use of a paragraph merging step reduces the number of paragraphs produced by 23% without affecting the performance of the system.

**Conclusions:**

The study shows that the presented system produces a coherent and logical structure for freely written nursing narratives and has the potential to reduce the time and effort nurses are currently spending on documenting care in hospitals.

## Background

Care documentation is important for supporting the continuity of care in hospitals. According to literature, nurses spend up to 35% (with an average of 19%) of their working time on documentation [1]. Naturally, if we can reduce the time that nurses spend on documentation, more time will be available for direct patient care.

To support tasks such as navigation, planning and statistical analysis, nurses in many countries are required to perform structuring of the information they write [2]. Such structuring approaches include the use of documentation standards, classifications and standardized terminologies [3]. However, this usually adds certain restrictions and requirements to the documentation process compared to writing the information in an unstructured narrative manner. In Finland, nurses are nowadays expected to structure the information they write by using subject headings from the Finnish Care Classification (FinCC) standard [4]. This includes selecting the correct subject headings and writing the associated information underneath. In this way, each subject heading forms a paragraph in the nursing note. As an example, if a nurse wants to write something about administrated wound care, he/she will first have to select an appropriate heading, e.g. “Wound”. FinCC consists primarily of two taxonomy resources, the Finnish Classification of Nursing Diagnoses (FiCND) and the Finnish Classification of Nursing Interventions (FiCNI), and both of these have a three-level hierarchy. For example, one branch in FiCND is: “Tissue integrity” (level 1), “Chronic wound” (level 2) and “Infected wound” (level 3). Another example, a branch from FiCNI is: “Medication” (level 1), “Pharmacotherapy” (level 2) and “Pharmaceutical treatment, oral instructions” (level 3). However, FinCC consists of more than 500 subject headings, covering both interventions and diagnoses. This makes it potentially challenging and time consuming for nurses to use since they are required to memorize, use and structure the information they write according to a large number of subject headings [5].

What we are aiming for is to develop a system that can assist nurses in selecting suitable subject headings and in structuring the text accordingly. We hypothesize that such a system has the potential to save time and effort required for documentation and ultimately free up more time for other tasks. We see two use-cases for such a system: One is where the system assists nurses in selecting appropriate headings when they write, in a suggestive manner, e.g., per sentence or paragraph; A second use-case is where nurses are allowed to write or dictate (by voice to text) in a fully unstructured narrative manner, without having to take into consideration the structure or the use of subject headings. Instead the system assigns subject headings afterwards and restructures the text into paragraphs. In this study we focus on the second use-case.

This is the continuation of a previously reported study that focused on assessing how an earlier version of the system performs on the level of sentences [6]. The main conclusion of that study is that a sentence classification model trained on semi-structured nursing notes can be applied on unstructured free nursing narratives without a substantial decline in accuracy.

This time we focus on paragraph-level assessment, where we also explore a post-processing step aimed at reducing the number of paragraphs initially generated by the system. To evaluate our system, a team of three domain experts (aka evaluators) conduct a manual evaluation to assess both the grouping of sentences into paragraphs and the correctness of the assigned headings. In addition we analyze the classification model in an attempt to identify conflicts between the actual use of the subject headings and the intended use according to the FinCC taxonomy.

At the core of our system is a text classification model based on a bidirectional long short-term memory (LSTM) recurrent neural network architecture [7, 8]. As training data we use a large collection of nursing notes from a Finnish hospital which contain subject headings and which are structured accordingly. Further, to acquire the type of narrative text that we would like to use as input to the system, without a bias towards a particular structure and subject headings, we made a set of nursing notes based on artificial patients that we use for testing.

### Related work

As we focus on classifying individual sentences, the work is closely related to other short text classification studies. However, most of the prior work focuses on texts collected from social media or other online sources [9–11]. Interestingly, Zhang et al. [12] conclude that the optimal text classification method is strongly dependent on the selected task, warranting domain specific research on this topic.

In the clinical domain, a common objective for text classification has been the automated assignment of ICD codes to the target documents [13–15]. For instance Xie et al. [16] use a neural model for mapping diagnosis descriptions extracted from discharge notes to the corresponding ICD codes. Similarly Koopman et al. [17] assign ICD-10 codes to death certificates, but limit the scope to various cancer types.

For cases where available training data is scarce, Wang et al. [18] propose a system for producing weakly labeled training data, where simple rules are initially used to label a large set of unlabeled clinical documents and these labels are subsequently used as training targets for machine learning based classifiers. The approach is evaluated on smoking status and hip fracture classification, but shows mixed results, with a rule-based baseline being the strongest model in some cases. As our training dataset inherently contains the used classification labels, we have not considered such weak supervision in our research.

To our knowledge the most recent systematic review on clinical text classification was conducted by Mujtaba et al. [19]. In addition to comparing the classification approaches utilized in different studies, the review focuses on the differences in the selected datasets. Their study indicates that along with the medical literature, clinical text classification research mostly focuses on pathology, radiology and autopsy reports, whereas other clinical documents such as nursing care records are far less studied. Moreover, the vast majority of the reviewed studies only evaluate their methods on English data, leading to Mujtaba et al. suggesting wider range of languages to be included in these studies.

As an additional note, Mujtaba et al. also conclude that deep learning methods are still relatively poorly studied in this domain. However, lately neural approaches have been suggested for a wide range of medical text classification purposes [20–22].

More related to our research are prior studies on clinical note segmentation. Denny et al. [23] present an approach for detecting section headers in clinical notes based on the free text. More precisely, they focus on history and physical examination documents where the goal is to identify and normalize section headers as well as to detect section boundaries. Li et al. [24] present a system that categorizes sections in clinical notes into one of 15 pre-defined section labels for notes already split into sections. Their approach relies on modelling the dependencies of consecutive section labels with Hidden Markov Models. In [25] coarse topics are assigned to the sections found in clinical notes. These topics are here seen as separate from the section headings used by the clinicians when writing, thus the section headings are considered as input to the classifier along with the free text.

A distinction between our study and the prior work is that we operate with an order of magnitude larger set of section labels. Additionally, we rely on semi-structured nursing notes as training data with the developed method subsequently being applied on unstructured notes. Thus, we do not utilize any prior knowledge about paragraphs/sections. Grouping the text into sensible paragraphs is instead a task for the presented system – together with assigning subject headings.

## Methods

Our ultimate goal is to develop a system that is able to automatically identify and classify, on sentence level, interventions and diagnoses mentioned in nursing narratives, and further capable of grouping the text into sensible paragraphs with subject headings reflecting their topics. In other words, we are aiming for a system that can let nurses simply write or dictate in a narrative manner without having to plan and structure the text with respect to paragraphs and subject headings. In pursuing this goal we have implemented a prototype system with a neural network-based text classification model at its core. In this section we describe the data and methods used in the implementation and evaluation.

### Data

The data set used for training is a collection of approximately 0.5 million patients’ nursing notes extracted from a university hospital in Finland. The selection criteria were patients with any type of heart-related problem in the period 2005 to 2009 and nursing notes from all units visited during their hospital stay are included. The data is collected during a transition period between two classification standards, the latter being the mentioned FinCC standard. This means that our training data contains a mixture of headings from these two. We only use sentences occurring in paragraphs with subject headings, which amounts to approximately 5.5 million sentences, 133,890 unique tokens and approximately 38.5 million tokens in total. We exclude all subject headings used less than 100 times, resulting in 676 unique subject headings, where their frequency count range from 100 to 222,984, with an average of 4,896. The individual sentences are used as a training example with the corresponding subject heading as the target class to be predicted. The average sentence length is 7 tokens^1^ and the average number of sentences per paragraph is 2.1. The data set is split into training (60%), development (20%) and test (20%) sets and further used to train and optimize the text classification model.

### Text classification model

The classification task is approached as a sentence-level multiclass classification task, where each sentence is assumed to have one correct subject heading (label). Our text classification model is based on a bidirectional short-term memory (LSTM) recurrent neural network architecture [7, 8]. The model receives a sequence of words as its input and encodes them into latent feature vectors (dimensionality 300). These vectors are subsequently used as the input for a bidirectional LSTM layer (dimensionality 600 per direction). As the final layer a fully connected layer with the dimensionality corresponding to the number of target subject headings is used. The word embeddings are pretrained with Word2vec [26]. The model is optimized for categorical cross-entropy with Adam optimizer [27], stopping early based on the development set performance. As machine learning tools we primarily use the Keras deep learning library [28], with TensorFlow library [29] as backend.

We want to emphasize that the focus of this paper is not to find the optimal text classification method and parameter settings for this task. This has instead been the focus of another study [30], where a range of different state-of-the-art and baseline text classification methods are tested and compared. Results from the mentioned study indicate that a bidirectional version of LSTM networks performs best when compared to other classification methods/models, including convolutional neural networks, support vector machines and random forests [31–33].

On the test set, when the classifier is allowed to suggest one subject heading per sentence, it suggests the correct heading for 54.35% of the sentences according to automated evaluation. When allowed to suggest 10 headings per sentence, the correct one is among these 89.54% of the time (see [30] for more details).

### Subject heading prediction and grouping into paragraphs

Since our prototype system relies primarily on a sentence-level classification model, it starts by classifying each sentence individually before grouping them into paragraphs. However, this might arguably be the opposite order of how a human would approach this task. The system’s operation can be described as a four-step process. **Step 1**: First the text is split into sentences. For this we rely on a combination of the NLTK tokenizers for Finnish [34] and a set of regular expressions tailored for the clinical text. **Step 2**: Next the classification model is used to classify each sentence individually and assign the top predicted heading (the one with the highest confidence value). **Step 3**: As a third step the sentences with the same assigned subject heading are grouped into paragraphs. **Step 4**: The fourth step focuses on merging paragraphs whose content and assigned headings are close to each other in terms of meaning. This fourth step is included to potentially reduce the number of paragraphs to more closely simulate how nurses document. Below we explain in more detail how this fourth step is done.

**Paragraph merging explained:** In the previous study [6], the evaluators reported that the system showed a tendency to assign subject headings with a high level of specificity, and sometimes even too specific to be practical. For example, for two or more sentences describing different aspects of pain management in the same nursing note, such as treatment and medication, the system would in some cases assign these to different subject headings, possibly headings of different level of specificity/abstraction. This meant that, in some cases, unnecessarily many unique headings, thus paragraphs, were assigned to each nursing note.

In an attempt to reduce the number of paragraphs created, to more closely simulate how nurses document, we have implemented an experimental post-processing step that enables the system to merge paragraphs (within a nursing note) that it finds to have similar subject headings. For this we primarily rely on the confidence values of the classification model, as well as extracted vector representations of each subject heading. The LSTM layer outputs 600 dimensional sentence encodings for both directions of the input sequence, resulting in 1200 dimensional vectors representations for the subject headings. These we use to calculate heading similarity by applying the cosine distance metric. See the “Data analysis” section for further description of these heading vectors.

First a paragraph-to-paragraph similarity matrix is formed reflecting how each paragraph would consider the subject headings from the other paragraphs (from step 3) as a likely candidate heading. To this end we define a simple asymmetric similarity function which measures how inclined a paragraph (source) is towards the heading of another paragraph (target) in the same nursing note. For each sentence in a given source paragraph we take the classifier’s confidence of the sentence belonging to the target heading and subtract the difference in the confidence between predicting the source heading and the target heading. The individual sentence scores are averaged and further summed with the cosine distance between the source and target headings and the relative size of the target paragraph (compared against the whole nursing note). The first component, relying on the confidence values of the classifier, describes how well the sentences fit in the target paragraph. The second component measures how semantically similar the compared paragraph headings are, more similar headings being more likely to be merged. The third component increases preference towards retaining the headings of the larger paragraphs. This scoring function produces values in the range 3 to minus 2. Note that it is not symmetrical.

To determine if two paragraphs are to be merged, we require that the similarity between these two paragraphs, in both directions, is above a given threshold. If the threshold is exceeded, the two most similar paragraphs are merged, keeping the heading of the paragraph with the lowest score out of the two. Subsequently the similarity matrix is recalculated, and the process is repeated until no paragraph pairs can be merged. We optimize this threshold on a sample of nursing notes from the test data where paragraph information and headings are removed. A threshold is found that enables the system to generate approximately the same number of paragraphs as in the original versions of these nursing notes.

### System evaluation

In this experiment the focus is on evaluating how the system performs at the intended task. Two versions of the system are manually evaluated, NoMerging and WithMerging, where the difference is that NoMerging only performs steps 1–3, while WithMerging also performs step 4. This comparison is done to see if the paragraph merging (step 4) can be done without reducing system performance according to the evaluators’ assessments. To perform the evaluation two domain experts with nursing background first evaluated the paragraphs individually. Then we consulted a third domain expert who provided a third opinion for the instances where the two disagreed. Finally the three of them agreed on the final consensus version which we report here.

The evaluation focuses on two aspects of the structured notes produced by the system: 1) The correctness of the assigned subject headings at paragraph level. Table 1 shows the classes used by the evaluators; 2) The quality of the formed paragraphs, i.e. sentence grouping. The classes used in this assessment are shown in Table 2.

**Table 1:** Classes used by the evaluators when assessing the headings assigned by the system

**Class**	**Description**
**1**	**Correct**: the subject heading suits the text in this paragraph.
**2**	**Partly correct**: the subject heading only suits some of the text, not all.
**3**	**Incorrect**: the subject heading does not seem to suit any of the text.
**4**	**Unable to assess**: unable to asses whether or not this subject heading is suitable.

**Table 2:** Classes used by the evaluators when assessing the paragraphs formed by the system

**Class**	**Description**
**a**	**Sensible grouping**: it makes sense to have these sentences grouped together as a separate paragraph based on their topic(s) (even if the subject heading may not fit).
**b**	**Inconsistent/problematic grouping – alt1**: one or more sentences in this paragraph would fit better in other paragraph(s) in this note.
**c**	**Inconsistent/problematic grouping – alt2**: one or more sentences in this paragraph do not belong in this or any of the other paragraphs in this note.
**d**	**Unable to assess**: unable to evaluate this paragraph.

**Fig. 1:**
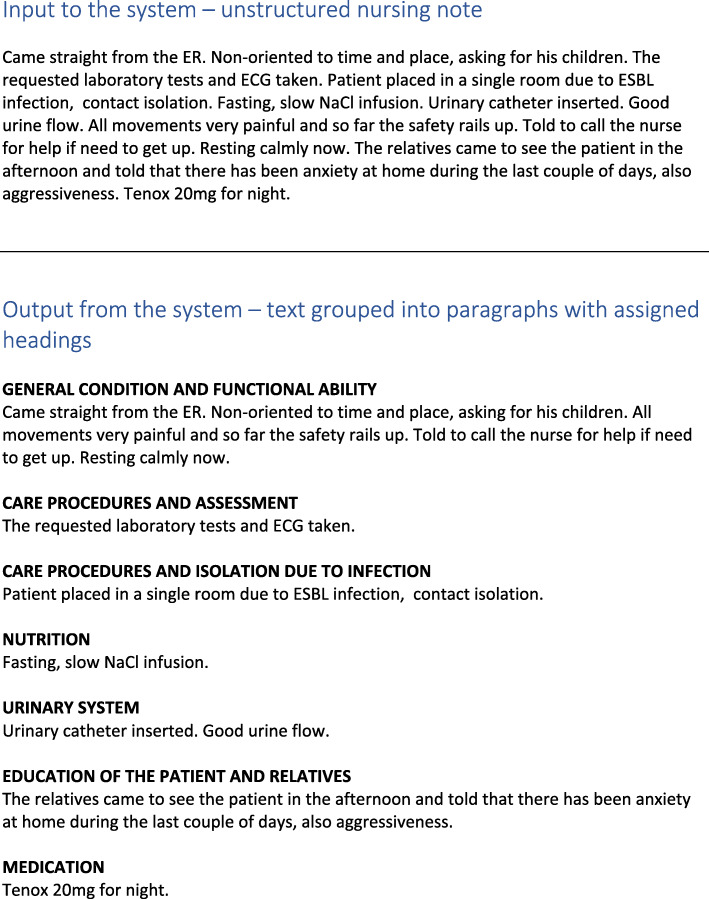
Nursing note example. **Top**: Without any particular structure or assigned subject headings. Input to the system. **Bottom**: Grouped into paragraphs with assigned headings. Output from the system. This has been translated from Finnish to English

The nursing notes from the training data have been planned, structured and written with subject headings in mind. One could argue that by simply removing headings and paragraph information, automated evaluation could be implemented. However, we found that the sentences here, which are structured under subject headings, have a tendency to be biased towards the topic of their headings, and sometimes their meaning can only be interpreted in the context of their headings. Also, this structuring forces the nurses to write very short and concise things, whereas when given the freedom to write in a narrative manner, more complex sentence structures are present. Thus, to obtain relevant nursing notes for evaluation of our use case – notes written in a free narrative style without planning for or considering the use of paragraphs and subject headings – we asked five domain experts with nursing background to write notes based on made up artificial patients. In total, 40 nursing notes, each note representing one day of provided care for a patient, were generated. The top part of Fig. 1 shows an example of one such nursing note.

These 40 nursing notes were fed to the two versions of the system, NoMerging and WithMerging. For evaluation purposes the output was stored as spreadsheets, one for each system, each containing both the original and the generated/structured version of each nursing note.

Statistical analyzes were performed to investigate differences in the manual evaluations of the two system versions (Pearson’s chi-squared test), as well as to see if there is a possible correlation between manual evaluations and the classification model’s confidence values (Spearman’s rho).

To gain some qualitative feedback on the system, we also asked the evaluators to answer the following open-ended questions:

1. Can you mention the main *strengths* that you found with the system(s)?
2. Can you mention the main *weaknesses* that you found with the system(s)?
3. Do you, or do you not, think that this kind of a system would be helpful when it comes to nursing documentation, and why?

### Data analysis

We hypothesize that the large amount of subject headings in the FinCC classification standard may cause confusion among the nurses in terms of what headings should be used in documenting the various aspects of the administrated care. Thus, to obtain a deeper understanding of the evaluated sentence classification model and the care documentation conventions of the nurses, we analyze the heading representations learned by the classification model – reflecting how they have been used – and how this may differ from their description and intended use based on FinCC.

The weights of the fully connected output layer of the trained classifier can be seen as semantic representations of the subject headings since the weights corresponding to a given heading define how strongly the heading is activated for a given input sentence, compared against other possible headings. Thus, two headings with similar weights will have similar probabilities of being assigned to a given input sentence. Inversely, under the assumption that the model has learned the classification task well, it can be hypothesized that if two headings have similar weights, the sentences assigned under these headings in the training data are also similar. Note that these representations are not based on the names of the subject headings, but instead on the actual sentences written under the headings.

Our main goal in this analysis is to verify whether we are able to find subject headings which are semantically similar according to our classification model, but far apart in the used FinCC taxonomy, or vice versa. This allows us to identify conflicts between the actual use of headings and their intended use according to the taxonomy. To measure the distances of the subject heading representations we simply calculate the cosine distance across all heading pairs.

The used FinCC classification standard is comprised of 3 top level categories: nursing diagnoses, nursing interventions and nursing outcomes, however the nursing outcome headings are not present in the used data. Both nursing diagnoses and interventions use a hierarchical structure with maximum depth of 3. To form a single tree, we connect the diagnoses and interventions categories with an artificial root node. This combined tree has a maximum depth of 4. To measure the distances of headings in FinCC we calculate the shortest path between the heading nodes in the tree. Although simple, this approach has shown strong performance in measuring concept similarities in other biomedical ontologies [35].

Once we have the two distances calculated for all subject heading pairs – cosine distance and distance in the tree – we rank each pair based on these two, resulting in two distinct rankings. The conflicting pairs that we select for further analysis are the ones being furthest apart according to these two rankings.

Since the nursing notes include the used subject headings as plain text, without containing the actual FinCC identifiers, we use strict string matching to map the headings to the corresponding FinCC concepts. This leaves us with 263 headings for this analysis out of the total 676 headings in our data set. The excluded headings either originate from the older classification standard or contain spelling variations.

## Results

In this section we first present the results from the system evaluation. Next we highlight some of the observations from the analysis of subject heading representations according to the classification model and the underlying classification standard.

### System evaluation results

This experiment provided insight into how the system performs at the intended task of assigning applicable subject headings and grouping sentences into paragraphs. Table 3 shows how well the assigned subject headings fit the text in the paragraphs. Table 4 reflects what the evaluators think about the integrity of the paragraphs formed by the system.

**Table 3:** Subject headings evaluation results. See Table 1 for an explanation of the classes

**Class**	**NoMerging**	*n*(tot=396)	**WithMerging**	*n*(tot=305)
**1**	70.45%	279	**71.15%**	217
**2**	14.65%	58	**16.72%**	51
**3**	14.14%	56	**11.80%**	36
**4**	0.76%	3	0.33%	1
**1+2**	85.10%	337	**87.87%**	268

**Table 4:** Paragraph (sentence grouping) evaluation results. See Table 2 for an explanation of the classes

**Class**	**NoMerging**	*n*(tot=396)	**WithMerging**	*n*(tot=305)
**a**	**79.55%**	315	79.02%	241
**b**	15.66%	62	**12.13%**	37
**c**	**3.79%**	15	8.52%	26
**d**	1.01%	4	0.33%	1

See Fig. 1 for an example showing a input note to the system (top) and the output (bottom) where the text is grouped into paragraphs with assigned subject headings.

Overall these results show that the system is able to provide suitable subject headings for about 71% of the paragraphs (class ‘1’). They also indicate that about 79% of the paragraphs formed are sensible (class ‘a’). By sensible paragraphs we mean that all the sentences within are related to the same topic and that none of them would fit better elsewhere in the corresponding nursing note.

When using NoMerging the number of paragraphs formed is 396, with an average of 9.9 per note (min=3, max=19). When using WithMerging, which also performs the paragraph merging step, the number of paragraphs is reduced by 23%, down to 305, with the average per note being 7.6 (min=2, max=17).

We also calculated how many of the formed paragraphs were consistent (class ‘a’) while also having a suitable subject heading (class ‘1’). The result is seen in Table 5 and shows that 66.67% (NoMerging) and 68.85% (WithMerging) of the paragraphs are both sensible and have a correctly describing subject heading assigned to them. These results show that the merging step results in basically no loss in performance.

**Table 5:** Results showing the percentage of sensible paragraphs (i.e. sentence groupings) with correct headings assigned

**Class**	**NoMerging**	*n*(tot=396)	**WithMerging**	*n*(tot=305)	
**1** and **a**	66.67%	264	**68.85%**	210	

Pearson’s chi-squared tests were performed to see whether there are statistically significant differences between the evaluation results of NoMerging and WithMerging based on 1) the subject heading correctness evaluations, and 2) the paragraph (sentence merging) quality evaluation results^2^. The evaluation of 1) does not seem to be dependent on what system version was used (*X*^2^ (2, N = 697) = 1.20, p = 0.55). However, there is a statistically significant difference between the two when looking at 2) (*X*^2^ (2, N = 696) = 8.12, p = 0.02).

It is possible that the confidence values of the classifier may provide some indication of paragraph correctness in that there is a correlation between the classifier’s confidence value for an assigned heading and the paragraph being correct according to the manual evaluation results. Using Spearman’s rho to compare the manual evaluation results of WithMerging with the classifiers confidence values for each paragraph’s assigned heading (average across sentences), we found there to be a negative correlation between classifier confidence values and the heading assignment ratings (Spearman’s rho = -0.42, “moderate”); as well as between classifier confidence values and the ratings of the quality of the formed paragraphs (Spearman’s rho = -0.29, “weak”).

Based on the open-ended questions posed to the evaluators, they reported the following.

1. As strengths they reported that the system does an overall (surprisingly) good job and usually provides good enough results.
2. Its main weakness and challenge is that people tend to write information about more than one topic into the same sentence. This sometimes makes it challenging for the system since it is tasked with classifying the entire sentence. They suggest that some sort of smart sentence splitting, which has the ability to split such sentences into two or more phrases, could help. Further, they also noticed that the basic sentence splitting performed by the system was not always correct, which sometimes resulted in the main message of a sentence being lost. One reported observation suggests that the system seems to perform worse on the more atypical and complex nursing notes.
3. On the question regarding whether or not the system could be helpful, they report that they think it could be (very) helpful since nurses would not need to consider where to write the information or what subject headings to choose. This would reduce time and effort required for nurses’ documentation duties. It is also suggested that this kind of system would work well when the documentation is done via dictation (speech to text). Another suggested consequence of using this system is that it could increase consistency in how headings are being used for similar information. However, it was also mentioned that having the ability to first select a set of subject headings can sometimes be helpful to remember what to report. The evaluators suggest that increased performance could be gained through fine-tuning the model for the different units at the hospital, possibly by limiting the pool of headings to select from.

### Data analysis results

**Fig. 2:**
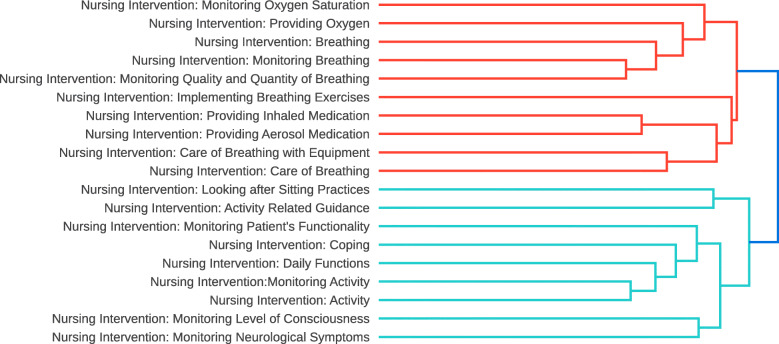
Heading Dendrogram. A subtree of the heading dendrogram formed with hierarchical clustering of the subject heading representations derived from the neural network classification model

Before looking in detail at the measured similarities between heading pairs, we examine the overall quality of the heading representations and the agreement between the two used rankings. To visually inspect the representations we form a dendrogram from a hierarchical clustering of the headings. Figure 2 shows an example subtree of the dendrogram with two high level clusters. The first one focuses on breathing, containing 10 headings overall, all of which are related to the topic. The second cluster contains 9 headings related to patient’s activity where most of the formed clusters are meaningful.

Although the heading vectors seem to offer good semantic representations, and the shortest paths have been used for measuring semantic similarity with other biomedical ontologies, there seems to be a strong disagreement between these two approaches. For instance the Spearman’s rho between the two formed rankings is only 0.12. To gain an insight into why these two rankings are heavily conflicted, we look at heading pairs that the classifier has identified as similar, but for which the corresponding distance in FinCC is high. It turns out that all top 1000 pairs with the largest difference in the ranks in this setting are pairs where one of the headings originates from the nursing diagnosis category and the other from the nursing interventions. The top conflicting pairs include headings such as “Nursing Diagnosis: Urinary Incontinence” – “Nursing Intervention: Treatment of Urinary Incontinence”, “Nursing Diagnosis: Changes in Oral Mucosa” – “Nursing Intervention: Basic Care of Oral and Other Mucosa” and “Nursing Diagnosis: Swelling” – “Nursing Intervention: Monitoring Swelling”.

To show that these headings are not similar according to our model only due to its incapability to distinguish the semantic differences between diagnoses and interventions, we look into the actual sentences written under these headings. For instance the “Nursing Diagnosis: Swelling” heading is assigned by nurses to sentences such as *Swelling of right arm.* and *Severe swelling of shins.* whereas “Nursing Intervention: Monitoring Swelling” heading contains sentences such as *Shins somewhat swollen.* and *Shins still swollen, feet not as much*. In fact, sentences such as *Legs swollen.* occur identically under both of these headings.

Similar trend can be seen by just looking at the most similar headings according to the classifier, ignoring the conflicting distance in the taxonomy. The most similar heading pair in the whole representation space is “Nursing Intervention: Providing Additional Nutrition” – “Nursing Intervention: Offering Supplements”. Both of these headings again contain identical sentences, such as *Renilon 1 can at 11 am* and *PreOp 2 cans, 6 o’clock*, yet these headings are not closely related in the FinCC taxonomy.

In classification tasks it is often the case that the amount of training data have a clear correlation with classification model performance, and that this can also be observed on the level of individual classes (i.e., subject headings in our case). However, based on the automated evaluation data, we do not observe a linear dependence between heading-specific model accuracy and the amount of training data for each heading (Pearson’s *r* = 0.04), probably due to the relatively large number of training examples for most classes. There is neither a linear dependence between accuracy and heading specificity (depth) in the FinCC taxonomy (Pearson’s *r* = 0.02).

## Discussion

Overall, the results suggest that the system is doing a relatively good job at the task of grouping nursing note text into paragraphs and labelling them with subject headings. According to the manual evaluation, shown in Table 5, 68.85% of the paragraphs formed by the WithMerging system variant are sensible and have been assigned subject headings that correctly describes the text therein. Even though the results are not yet perfect, we believe that the system could already be helpful by producing an initial structured version that the users can correct afterwards if needed.

We found that there is a correlation between the paragraph-level manual evaluation results and the classifiers confidence values for the assigned headings. Thus, for practical use, it could be helpful to the user to see these confidence values, for each paragraph and/or sentence, when assessing whether or not to retrospectively correct the initial structured version of a nursing note. As a future work we are considering training a separate model for the purpose of classifying the quality of formed paragraphs – e.g. as ‘good’ or ‘bad’.

In a previous study we conducted a manual evaluation of the same classification model as used here, but this focused on sentence-level evaluation instead of paragraphs [6]. In that study the evaluation was conducted on 20 nursing notes^3^ instead of 40, as in the present study. This previous evaluation showed that between 68.05% to 88.40% of the sentences had been assigned a suitable heading. In the present study, focusing on paragraphs, the results are similar, and equivalent to classes ‘1’ (71.15%) and ‘1+2’ (87.87%) in Table 3, accordingly.

These similarities are as expected for the NoMerging variant, since it merely groups together the sentences with the same assigned subject headings. More interesting is the observation that WithMerging has about the same performance as NoMerging (0.70 percentage point increase for class ‘1’ and 2.77 for class ‘1+2’). This indicates that the merging step performed by WithMerging does not result in less suitable headings being assigned to the paragraphs. We also observe that there is not a statistically significant difference between these evaluations. Further, by looking at Table 4, we see that the differences between the two system versions in terms of paragraph (sentence grouping) quality are small (e.g., only 0.53 percentage point decrease for class ‘a’ when comparing WithMerging to NoMerging). However, the Pearson’s chi-squared test shows that these differences can be considered as being statistically significant. We observe that the main differences between the assessments of the two system versions are found in classes ‘b’ and ‘c’, indicating that the paragraphs produced by WithMerging has fewer sentences which should be moved to other formed paragraphs within a nursing note (‘b’), but instead more sentences do not group well with any of the paragraphs it produces (‘c’). Whether this difference is of practical relevance is something that requires further investigation.

As already mentioned, this system has the potential to save nurses time and effort when it comes to documentation. As suggested by the evaluators, this system can be helpful when the documentation is performed via speech-to-text dictation – which alone has been shown to decrease documentation time [36, 37]. With a microphone being the main interface for the user (instead of a keyboard), it will be even more difficult to manually select and insert subject headings and structure the resulting text accordingly. Thus, with the use of such a system, nursing documentation could potentially be done, e.g., at the point of care and still produce nursing notes that follow the ruling documentation standard. The use of the proposed system also has the potential to increase the consistency in the use of subject headings for similar information and, as a consequence, improve the documentation quality.

Regardless of how the text is produced, a classification-based model like we use here could additionally serve as a reminder system that reminds the user about possible missing information in the nursing notes being written. For example, if a unit requires that certain topics should be mentioned in the nursing notes, the system will be able to detect (with a certain confidence) if something has not been reported yet. Similarly, the system could be used to notify users if a sentence already written under one heading/paragraph might better fit under another heading.

We did not put any limitations on the units and wards from where the nursing notes used in this study come from. Still, it is difficult to say how this system generalizes to the various units at the hospital. However, as mentioned in the answers from the evaluators, performance of such a system is likely to improve if it were to be tailored for individual units at the hospital. We believe that separate versions of the system could be used at the different units and wards at the hospital. In this way, the training data and what the classification model learns would more closely reflect the local documentation practices.

In the classification model used in our system, all training examples contained a sentence as input and a subject heading from the classification standard to predict as output. However, we have also observed that some of the text that the nurses document may not necessarily belong under a specific subject heading. Examples include meta information regarding the unit/ward, dates and names. As a future work we plan to also include such information as training examples for the model, and thus allow the system to suggest that some text does not need to be assigned a subject heading.

The paragraph merging step used here is rather primitive. Further, the system (WithMerging) is currently only allowed to merge the initially formed paragraphs. As a future work we plan to develop this merging algorithm further, where initially formed paragraphs may be split up to form new ones, and with the possibility of introducing new headings in the process. One idea could be to apply some sort of centrality-based algorithm.

The exploration of the heading representations formed by the classification model reveals a drastic discrepancy between the FinCC taxonomy and the actual use of the subject headings. The most prominent observation is that neither the classification model nor the nurses differentiate between diagnosis and intervention headings, but instead the same textual content is often documented under both variants of otherwise similar headings, e.g. “Swelling” (diagnosis) and “Monitoring Swelling” (intervention). Similar indistinguishable heading pairs can be detected within the main categories.

We believe these observations can be beneficial in developing future versions of FinCC as they provide a semi-automated method for identifying problematic taxonomy definitions based on a large collection of nursing notes, whereas the prior development has relied on small-scale questionnaires [38]. Since FinCC is derived from the international Clinical Care Classification (CCC) System [39], these issues are most likely not specific to FinCC, but also present in other patient care frameworks.

## Conclusions

In this study we have described the evaluation of a system aimed at assisting nurses in documenting patient care. The aim is to allow nurses to write the information they want to document without having to manually structure the text under subject headings which they select from a large taxonomy. Instead, the system automatically groups sentences into paragraphs and assigns subject headings. In 68.85% of the paragraphs formed by the system, the topics of the sentences are coherent and the assigned headings correctly describe the topics. Further, we show that the use of a paragraph merging step reduces the number of paragraphs produced by 23% without affecting the quality of the patient documentation, resulting in a more coherent outcome.

Finally, we show that interpreting the internal workings of the used neural classifier provides insights into the actual use of the subject headings in care documentation and can be used to pinpoint where the documentation practices deviate from the intended use of the care classification standards. Such observations can be utilized in improving the usability of the underlying clinical care taxonomy.

This study shows that the use of text classification applied to clinical nursing notes has the potential to reduce the time and effort that hospital nurses are currently spending on care documentation.

## Data Availability

The datasets generated and/or analysed during the current study are not publicly available due to their sensitive nature but more information are available from the corresponding author on reasonable request.

## Abbreviations

FinCC: Finnish Care Classification standard
FiCND: Finnish Classification of Nursing Diagnoses
FiCNI: Finnish Classification of Nursing Interventions
LSTM: Long Short-Term Memory recurrent neural network
ICD: International Classification of Diseases
CCC: Clinical Care Classification

## Footnotes

1 Space separated units.

2 Here we excluded classes ‘4’ and ‘d’ due to their low frequency (n <5).

3 These 20 nursing notes are a subset of the 40 ones used in the present study.
